# Wide Variation in Post-Void Residual Management after Urogynecologic Surgery: A Survey of Urogynecologists’ Practices

**DOI:** 10.3390/jcm10091946

**Published:** 2021-05-01

**Authors:** Marie-Louise Marschalek, Wolfgang Umek, Heinz Koelbl, Nikolaus Veit-Rubin, Barbara Bodner-Adler, Heinrich Husslein

**Affiliations:** Department of General Gynecology and Gynecologic Oncology of the Medical University of Vienna, Waehringer Guertel 18, 1090 Vienna, Austria; marie-louise.marschalek@meduniwien.ac.at (M.-L.M.); wolfgang.umek@meduniwien.ac.at (W.U.); heinz.koelbl@meduniwien.ac.at (H.K.); nikolaus.veit-rubin@meduniwien.ac.at (N.V.-R.); barbara.bodner-adler@meduniwien.ac.at (B.B.-A.)

**Keywords:** pelvic organ prolapse surgery, postoperative assessment, postoperative urinary retention, post-void residual, survey, voiding dysfunction, voiding trial

## Abstract

To date there is no standardized regimen or evidence-based practical guideline concerning post-void residual (PVR) measurement after urogynecologic surgeries. This survey aimed to evaluate current practice patterns and the approach taken among urogynecologists surrounding PVR measurement. An online survey was sent to members of several urogynecologic societies assessing pre- and postoperative management of patients undergoing urogynecologic surgery. A total of 204 urogynecologists from 21 countries participated in the survey. The vast majority of urogynecologists perform some kind of voiding trial to assess voiding function postoperatively. The cut-off values to perform catheterization, the methods of measurement, and the number of successfully passed voiding showed strong differences. Only 34.4% of the respondents consider routine PVR measurement after urogynecologic surgery to be evidence-based. PVR measurement after urogynecologic surgeries is widely performed and if pathological, it almost always provokes invasive treatment. However, there is a wide variation of implemented strategies, methods, and cut-off values. Scientific societies are challenged to devise a standardized regimen based on evidence for the management of urinary retention after urogynecologic surgery.

## 1. Introduction

The current life-time risk of undergoing any urogynecologic surgery is reported to be 20% for the female population [1,2]. After pelvic urogynecologic surgery, there is an elevated risk for voiding dysfunction or postoperative urinary retention (POUR), ranging between 2.5% and 43% [3,4,5,6]. The broader definition of voiding dysfunction includes any incomplete micturition. Post-void residual (PVR) is defined as the volume of urine left in the bladder after micturition and is not necessarily associated with complaints [7]. POUR is in most cases transient and the risk for prolonged retention—lasting 4 weeks or longer after surgery—is low [8]. Acute retention is generally associated with painful bladder and refers to the inability to pass urine despite a full bladder. It carries the risk of prolonged bladder distension and elevated intravesicular pressures, with subsequent myogenic and neurogenic damage, reflux and detrusor dysfunction, as well as urinary tract infections, pain, or even damage to the surgical repair of the prolapse [4,9,10].

Spontaneous or retrograde voiding trials confirm adequate voiding function and identify potential PVR after urogynecologic surgery. Therefore, screening for PVR after pelvic organ prolapse and urinary incontinence surgery is traditionally considered to be mandatory before patient discharge [4,11]. However, the methods of PVR evaluation vary across institutions and countries, and despite being widely used in multiple randomized trials [12,13,14], there is no standardized regimen or evidence-based practical guideline. Recent studies highlighted these variations [4,6,15].

Independent of existing practice variations, routine postoperative PVR measurement presents disadvantages. Overdiagnosis of clinically irrelevant urine residual is frequent and often prompts unnecessary invasive interventions such as catheterization. This kind of overtreatment is known to contribute to an increased risk of urinary tract infections, urethral trauma, patient discomfort, and prolonged hospital stay [16]. Moreover, the evaluation and management of POUR with frequent postoperative measurements might negatively affect patient satisfaction, in particular if catheterization is required. Furthermore, frequent measurements, additional visits and instrumentation of patients, consume material and human resources and increase health care costs, especially if the patient’s discharge is delayed. Previous studies showed that even low-cost routine interventions are responsible for substantial health care expenditures [17].

This survey aimed to evaluate current practice patterns and the approach taken among urogynecologists worldwide, regarding PVR measurement.

## 2. Materials and Methods

A survey among international experts in the field of urogynecology was conducted. An invitation for the online survey questionnaire was sent to members of the International Urogynecological Association (IUGA), European Urogynecology Association (EUGA), British Society of Urogynecology (BSUG), Urogynecologists in Canada, the National Urogynecology Working groups of Austria, Germany and Switzerland (AUB—Arbeitsgemeinschaft fuer Urogynaekologie und rekonstruktive Beckenbodenchirurgie, AGUB and AUG—Arbeitsgemeinschaft fuer Urogynaekologie und Plastische Beckenbodenrekonstruktion), between May and June 2020. The invitation was accompanied by a letter explaining the purpose of the study. Ethics approval was waived by the Institutional Review Board of the Medical University of Vienna, as the study did not include medical research involving human subjects, or identifiable human material and data according to the Declaration of Helsinki.

The survey consisted of 28 questions with an estimated time of 6 min to complete. It included demographic physician characteristics (age, present position, years in practice) and hospital characteristics (country, type, name, cases per year). Questions were then asked about practice patterns regarding pre- and postoperative management of patients undergoing pelvic organ prolapse and urinary incontinence surgery (catheter removal, duration of hospital stay, preoperative work-up). The main focus was on questions regarding postoperative PVR measurement (time, type of surgery, means of measurement, cut-off value, and management of persistent residual) and the approach taken with the reasons for practice patterns. In most cases, the respondents could select applicable answers from a list of options. Additionally, open questions were asked in order for the respondent to provide more information. Respondents were further given the opportunity to submit their comments in an additional field. The survey included questions that allowed multiple answers. The response percentages therefore might exceed 100%.

## 3. Results

A total of 204 urogynecologic experts from 85 centers in 21 different countries participated in the survey of which 48.3% (*n* = 98/203) were from public hospital departments, 46.3% (*n* = 94/203) from university hospital departments, and 20.7% (*n* = 42/203) from private practices (Table 1).

**Figure 1:**
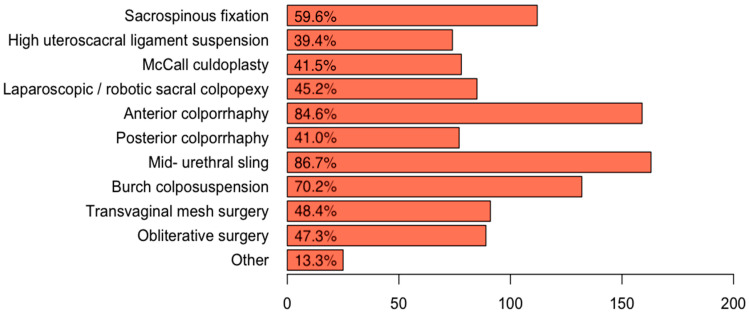
After which pelvic organ prolapse surgeries do you perform measurement of postvoid residual?

One hundred and sixty-nine participants fully completed the survey and answered all questions. After pelvic organ prolapse surgery, 12.4% (*n* = 25/201) removed the indwelling catheter on the day of surgery, 65.7% (*n* = 132/201) on the first postoperative day, and 17.9% (*n* = 36/201) on the second postoperative day. After incontinence surgery, 46.5% (*n* = 94/202) removed the indwelling catheter on the day of surgery, 43.6% (*n* = 88/202) on the first postoperative day, and 7.9% (*n* = 16/202) on the second postoperative day. The total duration of in-patient stay after pelvic organ prolapse surgery was less than 24 h in 13.4% (*n* = 27/201), 24–48 h in 47.3% (*n* = 95/201), 48–72 h in 27.4% (*n* = 55/201), and more than 72 h in 11.9% (*n* = 24/201). The total duration of in-patient stay after incontinence surgery was less than 24 h in 38.6% (*n* = 78/202), 24–48 h in 48% (*n* = 97/202), 48–72 h in 11.9% (*n* = 24/202), and more than 72 h in 1.5% (*n* = 3/202). A total of 70.9% (*n* = 144/203) of respondents always measured PVR after pelvic organ prolapse or incontinence surgeries, 26.6% (*n* = 54/203) measured depending on the surgery, and 2.5% (*n* = 5/203) never performed any measurement. A total of 10.2% (*n* = 19/187) measured on the same day of surgery and 51.9% (*n* = 97/187) measured PVR on the first postoperative day. A multitude of other possibilities were named in the “comment” field of the survey—“Depending on when the patient is going home”, “depending on when the catheter was removed”, “depending on the age and ability of self-catheterization of the patient”, “depending on the type of surgery”, “depending on the time of the first void”, and “depending on the time of removal of vaginal packing”. The most common time-point of PVR measurement mentioned in the “comment” field was “after catheter removal”. Among 86.7% (*n* = 163/188), mid-urethral sling surgery was the most common surgery, after which a PVR measurement was performed, followed by anterior colporrhaphy (84.6%, *n* = 159/188), Burch colposuspension (70.2%, *n* = 132/188), and sacrospinous fixation (59.6%, *n* = 112/188) (Figure 1).

The majority of respondents (91.6%, *n* = 174/190) performed PVR measurement by means of an ultrasound or bladder scan after a spontaneous void; 12.1% (*n* = 23/190) catheterized after a spontaneous void; and 7.4% (*n* = 14/190) performed a retrograde voiding trial. The cut-off value to perform catheterization was >150 mL in 25.5% (*n* = 48/188); >200 mL in 20.2% (*n* = 38/188); >100 mL in 16.5% (*n* = 31/188); and >1/3 of the total bladder volume in 19.1% (*n* = 36/188). Further answers in the “comment” field included “>300 mL”; “>400 mL”; “500 mL”; “700 mL”; “>1/2 of the voided volume”; “>1/2 of the total bladder volume”; and “void under 250 mL”. Concerning the number of successfully passed voiding trials required before discharge, 9.6% (*n* = 18/188) of the participants stated that the patients needed to successfully pass PVR measurements more than two times, 44.7% (*n* = 84/188) stated they needed to successfully pass PVR measurement twice, 44.1% (*n* = 83/188) stated once, and 1.6% (*n* = 3/188) stated never. In case of persistent PVR, the majority of physicians (62.1%, *n* = 118/190) would reinsert the indwelling transurethral catheter; 49.5% (*n* = 94/190) would instruct the patient to self-catheterize and 11.1% (*n* = 21/190) would perform a revision of the surgery. A few singularly mentioned options included electrostimulation; medication with cholinergic drugs and alpha-agonists; flip flow catheter and suprapubic catheter. One hundred seventy-five from a total of 195 (89.7%) respondents considered the measurement of PVR to be a necessary postoperative practice and 84 of 189 respondents (44.4%) declared that persistent PVR is a reason for prolonged hospital stay. The reasons for this approach were to avoid bladder overdistension injury with permanent bladder damage in 94.3% (*n* = 166/176); to avoid urinary tract infections in 52.8% (*n* = 93/176); to avoid pain in 42% (*n* = 74/176); and for patient comfort in 33% (*n* = 58/176). Among the 60 respondents who considered the practice to be unnecessary, 56.7% (*n* = 34) stated it caused an overdiagnosis of urine residual without any clinical benefit, 43.3% (*n* = 26) stated it caused frequent catheterization, and 38.3% (*n* = 23) stated it caused prolonged hospital stay without any clinical benefit. Further reported reasons were that it led to patient discomfort in 33.3% (*n* = 20) and that it consumed human resources and health care costs in 31.7% (*n* = 19).

## 4. Discussion

This survey aimed to evaluate the concept of PVR measurement, as well as the approach taken among urogynecologists. There is a wide variation in the management of PVR measurement, after pelvic organ prolapse and urinary incontinence surgery, in all participating institutions. This was consistent with previous studies that equally reported that PVR practices and voiding trials varied from clinician to clinician [4,6,15]. The vast majority of urogynecologists in various countries and institutions perform some kind of voiding trial to assess voiding function postoperatively.

The total duration of in-patient stay after pelvic organ prolapse surgery was mostly mentioned to be 24–48 h (47.3%). In 39.3% of cases, the duration is more than 48 h. Given that most acute postoperative retention is usually temporary, occurring either immediately postoperatively [18] or during the first few days after surgery, measurement of PVR might only be reasonable if the patient’s stay is anticipated to be short. The longer the hospital stay, the less necessary it is to measure PVR. The timing of removal of the indwelling catheter influences the incidence of acute postoperative urinary retention. This was demonstrated in a recent meta-analysis, which reported a significant difference in urinary retention incidence when urinary catheter removal was ≤6 h compared to >6 h [19]. With regards to the time when PVR is performed, most respondents (51.9%) stated to do so on the first postoperative day. However, there seem to be many aberrations and individualized decisions, as the physicians named a multitude of possibilities.

**Figure 2:**
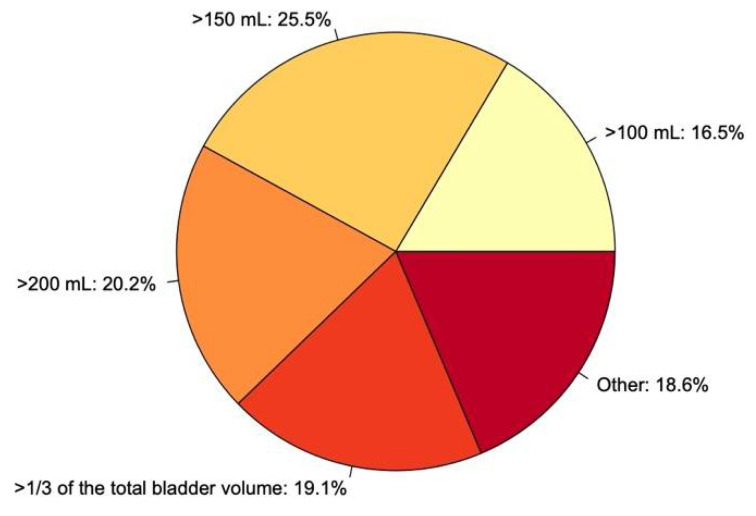
What is your cut-off value to perform catheterization?

In contrast to other studies, there was less variation in choosing the type of voiding trial among respondents of this survey [6,20]. Most urogynecologists (91.6%) perform the least invasive method of a voiding trial—the spontaneous voiding trial. Besides a broad range of definitions for abnormal post-void residual and for cut-off values to perform catheterization (Figure 2), there was also a difference in the number of successfully passed voiding trails that was required before discharge.

Several urogynecologists stated that the decision to catheterize is individual and dependent on pain, voiding difficulties or symptoms of incomplete voiding, as well as being dependent on preoperative values. These findings and heterogeneous responses highlight that there is no consensus of critical thresholds, and confirm that the cut-off values used by clinicians range significantly [4]. A 2021 systematic review of randomized controlled trials (RCT) investigating postoperative voiding trials, similarly summarized that the most significant inconsistency was the PVR criteria for a successful voiding trial, ranging from 100 mL to 500 mL [21]. A generally accepted cut-off level for urinary retention is not defined by the International Continence Society or any other national or international society [22]. This use of very different definitions, and the lack of standardization makes it difficult to compare study outcomes and made epidemiological studies impossible. Furthermore, it directly affected observed incidences of POUR and explained the wide variation of POUR incidence of 2−43% [4,6,23].

Additionally, our survey demonstrated that if women do not pass the voiding trial, they receive invasive treatment through repeated indwelling catheterization or clean intermittent catheterization, which commonly prolongs their hospital stay. Catheterization not only carries a risk of infection but is often considered the worst part of the surgical experience for patients [16,24].

Although PVR measurements after urogynecologic surgery are frequently performed among the participating urogynecologists and although most (89.7%, 175/195) stated that the measurements are a necessary postoperative practice, 65.6% (*n* = 128/195) do not consider this practice to be evidence-based. It is acknowledged that acute urinary retention risks damage the bladder, if not recognized and managed in time or if left untreated [25,26]. However, it is not clear what consequences asymptomatic PVR has for the bladder and when it is clinically relevant [9].

To identify and manage acute postoperative retention early enough, simple non-invasive techniques might be sufficient. A recent study evaluated if women are able to subjectively determine if they have emptied their bladder after a spontaneous void following urogynecological surgery to rule-out postoperative urinary retention by an objective voiding trial [27]. The negative predictive value of the subjective question regarding bladder emptying was >97%. This approach of simply asking the patient seemed to spare traditional voiding trials, without risking bladder damage due to acute postoperative retention. A recent RCT compared a backfill-assisted voiding trial with and without postvoid residual, after pelvic reconstructive surgery. Checking a PVR did not significantly affect voiding trial failure, postoperative duration of catheterization, UTI, or voiding dysfunction [28]. In another recent study, the incidence of postoperative urinary retention following benign gynecologic surgery was found to be similar to those with a strict voiding protocol and those with a liberal voiding protocol who were discharged with no voiding requirement [29]. The authors claimed that physicians intervened too quickly and that allowing the patient to void on their own schedule did not change the further course or elevate the risk of urinary retention.

This study has several limitations. As in any survey, the results underlay sampling, recall, and responder biases. All our results relied on the accuracy of responses. The veracity of the statements was unable to be checked. To limit sampling bias, multiple urogynecologic associations and societies in many countries were included. This made it possible to capture the practices of a large and diverse group of surgeons. Yet, although the invitation for the online survey questionnaire was also sent to IUGA members, the majority of the respondents were European. Furthermore, as invitations were sent to the members of the urogynecological societies by different means, we are unable to provide response rates, nor were we able to identify if the same physician filled out the questionnaire more than once.

## 5. Conclusions

This survey confirmed that there is an immense variation of implemented strategies, methods, and cut-off values, for the measurement of post-void residual after pelvic organ prolapse surgery, which is widely performed and almost always provokes invasive treatment. There is no widely accepted evidence-based standardized regimen. Future studies should consider comparing traditional voiding trials to non-invasive voiding strategies, by implementing a simpler evaluation such as subjective screening questions, patient education, and rescue mechanisms, if any voiding dysfunction occurs. The scientific societies should aim to seek a standardized clinical pattern, based on evidence for the prevention and management of postoperative urinary retention after urogynecologic surgery.

## Figures and Tables

**Table 1:** Demographic information of survey respondents.

Variable *n* = 204	*N* (%)
**Present position**	
Consultant	173 (85.2%)
Fellow	12 (5.9%)
Resident	18 (8.9%)
**Age**	
25–30 years	3 (1.5%)
31–40 years	35 (17.2%)
41–50 years	71 (34.8%)
>50 years	95 (46.6%)
**Country**	
United Kingdom	69 (33.8%)
Austria	33 (16.2%)
Canada	24 (11.8%)
Switzerland	22 (10.8%)
Germany	19 (9.3%)
France	9 (4.4%)
United States	6 (2.9%)
Italy	3 (1.5%)
Czech Republic	2 (1%)
Israel	2 (1%)
Serbia	2 (1%)
Slovenia	2 (1%)
Australia	1 (0.5%)
China	1 (0.5%)
Columbia	1 (0.5%)
Egypt	1 (0.5%)
Finland	1 (0.5%)
New Zealand	1 (0.5%)
Poland	1 (0.5%)
Sweden	1 (0.5%)
The Netherlands	1 (0.5%)
**Hospital**	
Public hospital	98 (48%)
University hospital	94 (46%)
Private practice	42 (21%)
Other	5 (2.5%)
**Years of clinical experience**	
0–5	10 (4.9%)
5–10	24 (11.8%)
10–15	32 (15.7%)
>15	138 (67.7%)
**Pelvic organ prolapse surgeries per year**	
0–5	12 (5.9%)
5–15	17 (8.3%)
16–30	36 (17.6%)
31–50	33 (16.2%)
50–100	59 (28.9%)
>100	47 (23%)

## Data Availability

Anonymized data will be shared on request from any qualified investigator.
